# Greater Patient Than Staff Satisfaction Scores for Electronic Consent

**DOI:** 10.7759/cureus.41810

**Published:** 2023-07-13

**Authors:** Shubhangi Fraser-Govil, Ahmed Elmowafy, Helen Pardoe

**Affiliations:** 1 Surgery, Princess Alexandra Hospital NHS Trust, Harlow, GBR; 2 Anaesthesia, Princess Alexandra Hospital NHS Trust, Harlow, GBR

**Keywords:** medico legal, patient satisfaction, staff satisfaction, digital exclusion, informed consent, electronic consent

## Abstract

Background

Informed consent is essential for surgical procedures, and using electronic consent (e-consent) has many benefits, including improved patient understanding and digitally enabled care. Following e-consent implementation at Princess Alexandra Hospital NHS Trust, Harlow, UK, we aimed to compare staff and patient satisfaction scores for the first time.

Methodology

Voluntary feedback was obtained via online questionnaires for patient and staff users. Average satisfaction scores were calculated, and comments were analysed using grounded theory and thematic analysis.

Results

Eight hundred and fifty-three counts of patient feedback and 36 counts of staff feedback were received. An average rating of e-consent for patients was 4.5 out of 5 and for staff was 2.8 out of 5. Fifty-one percent of patient comments and 25% of staff comments were positive. The main themes identified were information for patients, digital concerns, user experience, and functionality. There were conflicting positive and negative views from both groups within these themes.

Conclusions

E-consent enables informed consent for procedures, with greater satisfaction amongst patients than staff. The main factor that was appreciated by patients and staff is the ability of e-consent to facilitate fully informed consent.

## Introduction

Informed consent is a legal requirement when treating patients [1], and although the oldest evidence of written consent dates back to 1539 [2], it is a relatively modern construct. In the UK until 2015, the standard for informed consent was judged by the Bolam test, until it was replaced by the Montgomery principles, which state that patients should be made aware of any material risks [3].

Traditionally, paper-based consent forms have been used in hospitals for complex interventions such as surgery and are important medico-legal documents that contain vital information for patients, staff, and courts. However, error rates on hand-written consent forms can be as high as 45% [4], which is significantly reduced by using electronic forms [5]. E-consent follows the same general principles but has been found to improve patient understanding [6] and shared decision-making scores [7]. It supports the NHS long-term plan [8] and promotes sustainability. E-consent was introduced at Princess Alexandra Hospital NHS Trust, Harlow, a medium-sized UK district general hospital, in October 2022 to enable our local and national digital strategy [8], and our aim was to compare patient and staff experiences for the first time.

## Materials and methods

The trust began a project in March 2022 to phase out paper consent forms and replace them with an e-consent solution, Concentric® (Concentric Health, Cardiff, UK; https://www.concentric.health), which is a cloud-based application. Following user acceptance testing and development, end-user training began in July 2022, and in October, a soft rollout involving all patients undergoing surgery in the specialities of Obstetrics and Gynaecology and Orthopaedics began. Seven weeks later, e-consent was introduced trust wide. E-consent follows good practice guidelines [1] and enables two-stage consent, and forms can be shared electronically along with procedure-specific online information leaflets. The solution allows clinicians to take consent in person or remotely via a secure link and is configured for paediatric patients and patients lacking capacity.

Voluntary patient feedback was obtained via a system-based Concentric® questionnaire administered via email, which was developed by the Concentric® team. Staff feedback was obtained via a locally developed online questionnaire, which was disseminated internally. Questionnaires were independently designed to prevent performance bias. Both groups were asked to provide a satisfaction score out of 5 and optional comments, as per the appendices. Staff roles and specialities were recorded.

Patient feedback responses from the first 15 weeks were analysed, as theoretical saturation was reached. The staff survey was disseminated in January 2023, 10 weeks after the full rollout of the application, and was kept open for 10 weeks. Rating scores were calculated for patient and staff responses and staff type. Relevant comments were manually coded using grounded theory [9] by a single analyst, and independent validation was performed by a second analyst. Thematic analysis, performed manually, was used to draw conclusions.

## Results

In total, up to March 31, 2023, 11,380 episodes of consent were completed in our trust. Twenty-three percent of patients gave consent prior to the day of surgery, 10% of patients consented remotely, and 62% of consent forms were shared electronically with the patient. There was an average of 144 active staff users per week, and in the period in which patient feedback was analysed, 4438 consent forms were created.

Eight hundred and fifty-three patient questionnaires were returned with an average satisfaction rating of 4.6 out of 5. Staff feedback was obtained 36 times with an average score of 2.8 out of 5, which was consistent across different specialities and roles. 

There were 346 patient feedback comments, 177 of which were positive (51%), 21 were negative (6%), 91 were neutral (26%), and 57 were not related to e-consent. Out of the 36 staff comments, nine were positive (25%), 16 were negative (44%), and 11 were neutral (11%).

A total of 17 codes were identified, corresponding to four final themes: “information for patients,” “digital concerns,” “user experience,” and “functionality”, as per Table 1.

**Table 1:** Feedback themes and sub-themes

	Number	Percentage
Information for patients		
Good explanations	6	2.7%
Well informed	15	5.7%
Duplication of information	2	0.8%
Too much information	4	1.5%
More information needed	8	3%
Digital concerns		
Signature issues	36	13.6%
Technology issues	36	13.6%
Accessibility concerns	24	9%
Adapting to change	14	5.3%
User experience		
Time	30	11.4%
Interactions	15	5.7%
Less paper	4	1.5%
Private access	14	5.3%
Use of technology	4	1.5%
Functionality		
Ease of use	30	11.4%
Template issues	8	3%
Difficult to use	10	3.8%
Ability to follow-up	4	1.5%

Information for patients

This theme was mostly present in comments from patients but was also remarked upon by a few staff members. Codes within this theme were good explanations, well informed, duplication of information, too much information, and more information needed. This theme was generally positive, with patients appreciating the information provided about their procedure, with comments such as:

“Patients are coming to theatres well informed.”

“Well explained and very informative does not need any changes.”

However, some patients felt there was too much information provided, and other patients felt they weren’t provided with enough information.

“A little less wordy would be good.”

“More information in consent, risk versus equivalent daily risk.”

Digital concerns

The codes under this theme were the most numerous. Signature issues were commented on by staff and patients, difficulty using a mouse or a finger to sign was included, and signatures were not recognisable. Technical difficulties, including connectivity, were an issue for both groups.

“The Wi-Fi connection was poor, so the process was difficult.”

“Difficult to master a mouse for signatures for a person of my age.”

“I completed the form on a phone and found the signature difficult - fat fingers and small box!”

“A lot of our patient demographic are elderly, so either don’t use a smartphone or find it difficult to sign on the screen. Furthermore, the general 4g connectivity is poor across the hospital, so when patients are using their own devices to scan the QR code, it still may not work due to poor internet connection.”

Accessibility was a concern, with patients and some staff noting they were not able to interact well with the electronic system or they thought others would not be able to. 

“Not all patients have email or mobile phone.”

“Elderly patient found it very difficult to sign this consent device with a disabled shoulder and restricted mobility. I took several attempts to achieve.”

“Satisfied with the digital consent process but not sure it would be suitable for everyone taking into account patients with various disabilities.”

“It was on a computer screen in a position where I couldn’t read it. Too far away for me to see the text with normal sight and too far for reading glasses! I should have been able to read the consent before being seated for the treatment. As it was, the consultant briefly read through aloud to me.”

Adapting to change was mostly brought up by staff, issues including staff training and difficulty managing the transitionary period.

“E-consent is helpful, but not many use it, which leads to a lot of confusion regarding consent forms.”

“All clinicians should have completed necessary training before e-consent went live, still having issues with clinicians that are on extended leave.”

User experience

The impact of e-consent on both saving time and taking too much time was a recurring topic for both groups. Some patients felt they were not provided adequate time for the consent process.

“When used by trained staff can save time.”

“Takes 10 times longer at the present time than paper consenting.”

“I was well satisfied the process was efficient, well explained, and clearly saved valuable time.”

“A bit rushed. Could have done this before in with consultant.”

Patients commented on their experience of staff interacting with the system, in both positive and negative ways.

“The poor lady trying to use the software had a nightmare; it was impossible to conclude the registration; I felt for her.”

“Doctor reading from the screen does not instil confidence in knowledge of procedure.”

Some patients appreciated that e-consent was utilising modern technology and using less paper, but this was sporadic in the feedback.

“Nothing to improve so much better signing a consent and received your paperwork straight to your email and no waiting around for letters.”

Functionality

Generally, patient feedback commented on the ease of electronic consent and the efficiency, but area patients wanted improvement in being able to follow up on their information.

“Was not sure if consent sent, so an acknowledgement would be good.”

“I was very impressed with this new way to capture patient consent and said so to the doctor during my consultation. I think it’s an efficient setup and much better to be able to add this on to a patient’s electronic records.”

Staff feedback was quite negative on the usability of the system, highlighting issues with the pre-defined templates and difficult use in clinical practice.

“There are a lot of procedures that are not pre-populated, and it takes a lot of time to add all the information in along with the patient-specific risks.”

“Not fit for purpose. The concept is good, but the IT in the trust is atrocious and doesn’t support it. So cumbersome and missing so many complications. Leads to delays unnecessarily.”

## Discussion

It is clear that patient satisfaction is high with e-consent, and factors that patients particularly appreciate are the usability of the system, the speed of use, and that they are better able to give informed consent. Staff also felt more confident that patients gave fully informed consent. Staff satisfaction ratings were lower, and comments were generally more negative. Staff highlighted issues with the system and that it can take more time. The major issue frequently mentioned in patient feedback was difficulty with electronic signatures, which staff also commented on. Electronic signatures are known to be an issue in healthcare [10] but are medico-legally approved [11]. The legibility of hand-written signatures can also be poor [12] and was not studied here.

Strengths of this qualitative research include the large number of responses received, and as all feedback was clearly highlighted as anonymous, we were able to receive honest feedback displaying both positives and negatives. The main limitation is that there may be an element of response and non-response bias, for both groups.

Response bias may be a reason for lower staff satisfaction; as generally, NHS staff responses to surveys are low [13], and it is known that higher response rates can be seen when there is very low satisfaction, with more middling views not being recorded [14]. Furthermore, change can be difficult for staff [15], as it conflicts with the need for stability, and different characteristics can make individuals more resistant to change [16]. This may also be a reason for the lower staff satisfaction seen here. However, recognising patient benefits and having long-term support and commitment to the change can make changes more acceptable and successful in the longer term [17,18].

Non-response bias for patients may be present, as patient feedback was obtained from questionnaires sent to the email address to which consent forms were shared, so we may be missing feedback from those who did not provide an email address. Therefore, there may have been an element of digital exclusion [19,20] in the survey responses. Groups particularly affected by this were not identified in this study, but they may be those who are older, an ethnic minority, or with physical or mental health disabilities [21]. As the survey was only available in English, this would have led to the exclusion of non-English speakers, and further research could include surveys in other languages to obtain a wider range of feedback.

Literature on patient and staff satisfaction with e-consent in clinical practice is nascent, but some studies have shown a preference for e-consent [22], and higher shared decision-making scores have been demonstrated [7,23]. E-consent has been widely used in medical research, and studies have shown similar findings, in which e-consent participants are satisfied and can have a better understanding of information, but there are issues with using technology and authentication [24].

E-consent has enabled the organisation to reduce the paper used and become more digitally enabled. There are still improvements to be made, such as with connectivity and increasing two-stage consent [25]. Further studies could evaluate if there are any patient or clinician characteristics that affect satisfaction scores.

Ultimately, e-consent is a tool to enable informed consent [26] and cannot prevent poor experiences or consultations. However, when used well, patients feel more empowered and are better informed, so greater availability of healthcare technology may be a way to improve care.

## Conclusions

In the post-implementation of e-consent at Princess Alexandra Hospital NHS Trust, Harlow, a medium-sized UK district general hospital, patient satisfaction was higher than staff satisfaction. Issues with technology and electronic signatures were frequently mentioned as adverse factors, and there was conflicting feedback about usability and timeliness between patients and staff. The major assurance that e-consent gave to both staff and patients was the ability of the system to ensure that patients gave fully informed consent about their procedure.

## Appendices

Patient survey

Concentric is the digital consent application being used at The Princess Alexandra Hospital NHS Trust and aims to support you in making decisions about treatment. You have received this survey link from your clinician because you have given your consent to treatment digitally, either in person with your clinician or remotely. Your responses will not impact your care. To find out more about Concentric, you can visit the Concentric website.

·        How satisfied were you with the Concentric digital consent process?

o   1 (Very Dissatisfied)  2  3  4 5 (Very Satisfied)

·        Do you have any other comments on your experience? What is the one thing that could be improved on?

o   This could be something that you particularly appreciated, an opportunity for improvement to the consent process, or a modification to the Concentric application.

Staff survey

Many thanks for completing this short questionnaire about our new E-consent system. We have been live trust-wide since 5/12/22 and looking to evaluate staff feedback for research purposes. All answers will be anonymous. If you have any further questions or require technical help, please feel to email

·        What is your role?

o   Nurse, ODP, Healthcare Assistant, Consultant, Doctor (non-consultant), Midwife, Other

·        What is your speciality?

o   General Surgery, Orthopaedics, Obstetrics & Gynaecology, Ophthalmology, Dermatology, MaxFax, ENT, Urology, Radiology, Cardiology, Gastroenterology, Colorectal Surgery, Other

·        How many stars would you give e-Consent out of 5?

o   1 (poor), 2, 3, 4, 5 (excellent)

·        Do you have any specific comments?
